# Predicting radiofrequency thermocoagulation surgical outcomes in refractory focal epilepsy patients using functional coupled neural mass model

**DOI:** 10.3389/fneur.2024.1402004

**Published:** 2024-08-23

**Authors:** Tianxin Cai, Yaoxin Lin, Guofu Wang, Jie Luo

**Affiliations:** ^1^School of Biomedical Engineering, Shenzhen Campus of Sun Yat-sen University, Shenzhen, China; ^2^Key Laboratory of Sensing Technology and Biomedical Instrument of Guangdong Province, Guangdong Provincial Engineering and Technology Center of Advanced and Portable Medical Devices, Sun Yat-sen University, Guangzhou, China; ^3^Department of Functional Neurosurgery, First People’s Hospital of Foshan, Foshan, China

**Keywords:** coupled neural mass model, stereoelectroencephalography, focal epilepsy, functional connectivity, parameter identification

## Abstract

**Objective:**

The success rate of achieving seizure freedom after radiofrequency thermocoagulation surgery for patients with refractory focal epilepsy is about 20–40%. This study aims to enhance the prediction of surgical outcomes based on preoperative decisions through network model simulation, providing a reference for clinicians to validate and optimize surgical plans.

**Methods:**

Twelve patients with epilepsy who underwent radiofrequency thermocoagulation were retrospectively reviewed in this study. A coupled model based on model subsets of the neural mass model was constructed by calculating partial directed coherence as the coupling matrix from stereoelectroencephalography (SEEG) signals. Multi-channel time-varying model parameters of excitation and inhibitions were identified by fitting the real SEEG signals with the coupled model. Further incorporating these model parameters, the coupled model virtually removed contacts destroyed in radiofrequency thermocoagulation or selected randomly. Subsequently, the coupled model after virtual surgery was simulated.

**Results:**

The identified excitatory and inhibitory parameters showed significant difference before and after seizure onset (*p* < 0.05), and the trends of parameter changes aligned with the seizure process. Additionally, excitatory parameters of epileptogenic contacts were higher than that of non-epileptogenic contacts, and opposite findings were noticed for inhibitory parameters. The simulated signals of postoperative models to predict surgical outcomes yielded an area under the curve (AUC) of 83.33% and an accuracy of 91.67%.

**Conclusion:**

The multi-channel coupled model proposed in this study with physiological characteristics showed a desirable performance for preoperatively predicting patients’ prognoses.

## 1 Introduction

Epilepsy is a serious neurological disorder that affects the sensory, motor, and autonomic functions of patients to varying degrees (1). Focal epilepsy is the result of specific local lesions within brain networks (2, 3). Approximately one-third of epilepsy patients are drug-resistant (4), meaning that they cannot achieve seizure control despite taking anti-epileptic drugs (5, 6). Epilepsy surgery is recommended for drug-resistant focal epilepsy by removing the brain tissue responsible for the seizures (7). During the preoperative evaluation, clinicians use a variety of medical techniques to identify the surgical area, known as the epileptogenic zone (EZ). Stereoelectroencephalography (SEEG) is currently the gold standard for localizing EZ in clinical practice (8, 9). Specifically, multiple-depth electrodes are placed in different brain regions in a three-dimensional manner and electrical signals during ictal and interictal periods are recorded precisely. However, the analysis of SEEG signals and the judgment of EZ largely rely on the experience of clinicians, which limits the accurate identification of the EZ. Notably, misjudgment greatly affects preoperative decisions, leading to ineffective epilepsy surgery (10) and preventing patients from achieving seizure freedom (SF) after surgery. Follow-up studies on patients who underwent radiofrequency thermocoagulation surgery indicate a success rate of achieving SF about 20–40% (11–13). Therefore, this study aims to use model simulation to predict surgical outcomes based on preoperative decisions, provide a reference for clinicians to validate and optimize surgical plans, and ultimately help improve the success rate of radiofrequency thermocoagulation surgery.

It is well-known that epilepsy is a brain network disorder (14). The traditional concept of epileptogenic foci believes that seizures originate from relatively localized brain regions (7) while existing studies have found that the distribution of epileptogenic foci may be relatively widespread. In most cases, seizures initiate from several different brain areas, discharging simultaneously or in rapid succession (15). Some researchers proposed that the brain regions that generate seizures (i.e., the epileptogenic network) are responsible for the earliest onset of seizures, generating high-frequency oscillations and subsequently triggering low-frequency oscillations in the propagation network (16). In this context, many studies in recent years have focused on connectivity analysis of brain networks instead of traditional single-channel electroencephalogram (EEG) analysis to calculate epileptic indexes. As a result, the research perspective is expanded from temporal patterns to spatiotemporal patterns.

Brain connectivity can be quantified using different metrics, including anatomical structural connectivity, statistical functional connectivity or effective connectivity. Specifically, structural connectivity is derived from diffusion tensor imaging (DTI) and fiber tractography and provides high spatial resolution (17). Functional connectivity is calculated from empirical data like EEG and includes classical measures such as correlation, information entropy, phase synchronization, and Granger causality. Among these connectivity measures, directed functional connectivity is known as effective connectivity. Compared to structural connectivity, functional connectivity is limited by the spatial sampling rate of empirical data and cannot study the entire brain network. However, surgical centers in developing countries rarely have structural connectivity due to limited scanner availability and scanning time (18). Among functional connectivity measures, partial directed coherence (PDC) based on Granger causality, provides a frequency-domain measure (19, 20) that can consider and adjust the effect of indirect connections (21), making it suitable for large-scale network connectivity analysis. But it is worth noting that PDC can only provide relative information of coupling strength, and its absolute values cannot be directly interpreted (22, 23).

In recent years, the integration of neural computational models with connectivity networks has emerged to investigate the complex neurophysiological activities of large-scale brain networks. Neural mass models (NMM) at the mesoscopic level are physiologically relevant because neurons within specific brain regions are organized into neural mass. Jansen and Rit (24) developed a NMM with three subpopulations, including excitatory pyramidal neurons, excitatory interneurons, and inhibitory interneurons. Although Jansen’s model has been widely used, it is not suitable for epilepsy research due to its inability to capture high-frequency dynamics of signals. Wendling et al. (25) developed a new model by adding a fourth subpopulation of fast inhibitory interneurons based on Jansen’s model, which improved the dynamics of the model system and was able to describe rapid EEG activity. Besides, Jirsa’s team extended NMM to neural field models and conducted a series of studies incorporating multimodal data like DTI (17, 26–33). A full-brain-network simulation platform called The Virtual Brain (TVB) and a patient-specific model called Virtual Epileptic Patient (VEP) were developed by their team. Although TVB and VEP can simulate epileptiform discharges and have been applied in various clinical aspects, their modeling requires complex neuroimaging examinations and extensive computational resources (18).

In addition to structural connectivity, functional connectivity is also a method used to couple models, but related studies are limited. Taylor et al. (34) introduced a bi-stable system model as a node and established a coupled network model for patients with epilepsy by using the functional connectivity matrix of electrocorticography signals to couple node models. The authors simulated surgical interventions by altering the connectivity matrix and compared the escape time of simulated signals after actual and random resections to predict surgical outcomes. Yang et al. (35) computed adaptive directed transfer function from SEEG signals as functional connectivity matrix, coupling multiple subsets of Wendling models to form a network model. They performed virtual surgery by removing specific subsets from the model and found that the simulated signals had fewer spikes when removing subsets within the EZ compared to removing subsets outside the EZ, thereby predicting good surgical outcomes for these patients. However, the above brain network models only consider the connectivity information, and the excitatory and inhibitory characteristics of the model are set as constants. In fact, the excitatory and inhibitory dynamics of neural networks are not constant during epileptic seizures (36), and disruption of the dynamic balance may be a cause of excessive excitation in epileptic networks (37). Therefore, it is worthwhile to study the dynamics of excitation and inhibition while taking connectivity into account.

In this study, a virtual surgery study was performed using SEEG data obtained from 12 focal epilepsy patients who underwent radiofrequency thermocoagulation. Firstly, PDC was calculated based on the SEEG signals as the coupling matrix, which reflects the connectivity of the brain network. For each contact of SEEG, a model subset of Wendling’s NMM was built, and the PDC matrix was used to couple subsets together to construct a coupled model. By fitting the coupled model to the real SEEG signals, the time-varying model parameters of excitation and inhibitions were determined. Thereafter, virtual surgery was performed on the coupled model (i.e., altering the connectivity of the model) depending on the specific contacts destroyed during radiofrequency thermocoagulation or selected randomly. The simulation of the postoperative model incorporated time-varying model parameters of the excitation and inhibitions. The results showed that the comparison of simulated signals from postoperative models after clinical and random virtual surgery were able to reflect the actual surgical outcomes.

## 2 Materials and methods

### 2.1 Clinical data and preprocessing

The clinical data for this study was obtained from First People’s Hospital of Foshan. Records from 22 patients with focal epilepsy who underwent SEEG at the Functional Neurosurgery Department between January 2018 and April 2024 were reviewed. Patients were excluded due to no presurgical evaluation and radiofrequency thermocoagulation treatment (two patients), unavailable SEEG recording with at least one seizure (one patient), unavailable magnetic resonance imaging (MRI) and computed tomography (CT) scans (one patient), <1 year of follow up (six patients). Twelve patients met inclusion criteria and were retrospectively studied. The 12 included patients were recorded as patients 1 to 12, and their specific clinical information is presented in Table 1.

**Table 1:** The clinical information of 12 patients with focal epilepsy obtained from Foshan First People’s Hospital.

Patient	Gender	Age (years)	Seizure type	MRI findings	Sampling rate (Hz)	Number of contacts	Location of surgery	Anti-seizure medication	Postoperative follow-up (months)	Surgical outcome (Engel Class)
1	F	5	FAS	N	256	108	PreCG.L	LD (discontinue)	72	SF (I)
2	F	36	FIAS	TLL.R, HS.R	4,096	160	HH.R	Oxc, LD	71	NSF (III)
3	F	39	FIAS	HS.B	512	77	HH.R AMY.R	Lev, Oxc	54	SF (I)
4	M	18	FIAS	SGMH.B	4,096	198	SGMH.L	Lev, Oxc, LD	44	SF (I)
5	M	40	FIAS	N	4,096	177	HH.R AMY.R	Car, TT, SV	42	NSF (II)
6	F	3	FAS	FLD.B, PLD.B	4,096	145	HT.R	Per, Lac, LD, TT	43	NSF (III)
7	M	23	FIAS	N	4,096	187	INS.R	Oxc, Lev, Clo, LD, SV, Per	36	NSF (III)
8	M	12	FIAS	N	4,096	121	CG.L	TT, Per, LD, Clo	35	SF (I)
9	M	25	FIAS	SGMH.L	4,096	216	HH.L AMY.L	Lev, TT	31	SF (I)
10	M	10	FAS	N	4,096	72	PreCG.L PoCG.L	Oxc, LD, SV, Per	16	SF (I)
11	M	22	FIAS	N	4,096	179	INS.R OPE.R	Lev, TT	26	NSF (II)
12	M	27	FIAS	HS.L	4,096	132	PL.L	Oxc, LD, SV, Lev	20	NSF (III)

F, female; M, male; N, negative; FAS, focal aware seizure; FIAS, focal impaired awareness seizure; L, left; R, right; B, bilateral; TLL, temporal lobe lesion; HS, hippocampal sclerosis; SGMH, subependymal gray matter heterotopia; FLD, frontal lobe demyelination; PLD, parietal lobe demyelination; PreCG, precentral gyrus; HH, hippocampal head; AMY, amygdala; HT, hippocampal tail; INS, insula; CG, cingulate gyrus; PoCG, postcentral gyrus; OPE, operculum; PL, parietal lobe; LD, lamotrigine dispersible; Oxc, oxcarbazepine; Lev, levetiracetam; Car, carbamazepine; TT, topiramate tablets; SV, sodium valproate; Per, perampanel; Lac, lacosamide; Clo, clonazepam; SF, seizure-free after surgery; NSF, non-seizure-free after surgery.

In the presurgical evaluation, specialist doctors combined clinical symptoms, non-invasive electrophysiology, and imaging findings to comprehensively determine the potential EZ. SEEG depth electrodes were then implanted into the identified brain regions. In the 12 included patients, the number of implanted depth electrodes ranged from 7 to 15, each including 6 to 18 contacts (Alcis, France or HKHS Health care, China). Next, a SEEG acquisition device (American Nicolet EEG 256-channel amplifier) was used to record the patients’ SEEG signals. Clinicians manually divided electrode contacts within EZ by observing the SEEG signals from each channel. The identified brain tissue near these contacts was then targeted for radiofrequency thermocoagulation surgery. The radiofrequency thermocoagulation was performed without anesthesia. The thermocoagulation foci were created by using an RF lesion generator equipment, model R-2000B M1 (Beijing Neo Science Co., Ltd., Haidian, Beijing, China). The lesions were made by using a current with the power controlled at 3–3.5 W, applied for 30–50 s. The thermocoagulation temperature reached 78–82°C, causing irreversible damage to the tissue in the range of 5–8 mm. After surgery, clinicians followed patients for at least 1 year to record their postoperative prognosis. This retrospective study was approved by the Medical Ethics Committee of First People’s Hospital of Foshan.

The corresponding brain regions of the implanted electrode contacts were identified by automatic contact localization software (38) using MRI and CT data. For the patients’ SEEG data, 30 s segments of SEEG signals before and after the seizure onset were intercepted according to observation of signal channels and annotations by clinicians. Two segments were analyzed for each patient (patient 6 had only one segment). Additionally, signals from each channel were observed and channels with artifacts were removed. Subsequently, the signals were filtered using a 50 Hz power line frequency filter and a Butterworth band-pass filter with a cut-off frequency of 0.2–120 Hz. A unipolar reference was used for data analysis, and the reference electrode was the ipsilateral ear. Finally, to standardize the sampling rate of signals from different patients and facilitate subsequent data processing, all data segments were downsampled to 256 Hz.

### 2.2 Calculation of functional connectivity matrix

**Figure 1:**
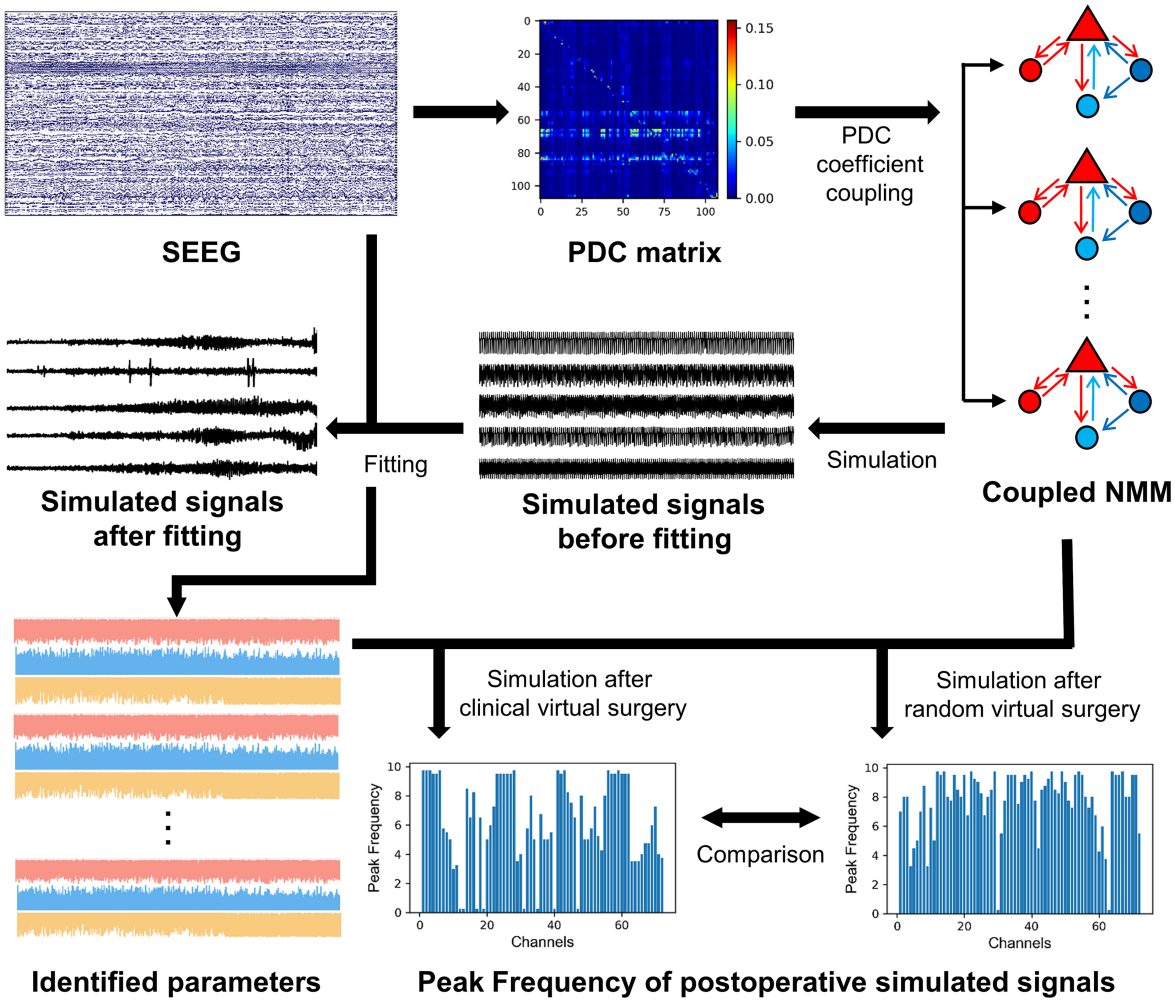
The overall flowchart of this study.

Figure 1 provides an overall flowchart of this study. To construct a personalized brain network model for patients, it is necessary to analyze their brain connectivity status. The PDC method from functional connectivity analysis was employed to measure the connectivity between different brain regions. PDC is based on a multichannel autoregressive (MAR) model that utilizes past information from multiple channels to predict the activity of current channel. During the fitting process of MAR model, the model coefficients and white noise residuals were estimated. The MAR model can be represented by the following equation:


$$\left[\begin{array}{c} x_{1}\left(n\right)\\ \vdots \\ x_{m}\left(n\right) \end{array}\right]=\overset{p}{\underset{i=1}{\sum }}C_{i}\left[\begin{array}{c} x_{1}\left(n-i\right)\\ \vdots \\ x_{m}\left(n-i\right) \end{array}\right]+\left[\begin{array}{c} w_{1}\left(n\right)\\ \vdots \\ w_{m}\left(n\right) \end{array}\right]$$

$$C_{i}=\left[\begin{array}{ccc} c_{11}\left(i\right) & \cdots & c_{1m}\left(i\right)\\ \vdots & c_{ij}\left(i\right) & \vdots \\ c_{m1}\left(i\right) & \cdots & c_{\mathrm{mm}}\left(i\right) \end{array}\right]$$

where *x*_1_(*n*), …, *x_m_*(*n*) represent the signal sequences of *m* channels at the time *n*, *w*_1_(*n*), …, *w_m_*(*n*) are the white noise residuals, *p* denotes the order of the model coefficients, **C***_i_* represents the coefficient matrix of dimension *m* for the order *i*, and the matrix element *c_ij_* signifies the influence of channel *j* on channel *i*.

Afterward, the coefficient matrix **C***_i_* is subjected to Fourier transform, resulting in the frequency-domain coefficient matrix **A**(*f*) with *m* dimension. The formula is as follows:


$$A\left(f\right)=\overset{p}{\underset{i=1}{\sum }}C\left(i\right)e^{-j2\pi fi}$$

Finally, the element *PDC_ij_* in the PDC matrix can be obtained as follows:


$$\bar{A}\left(f\right)=I-A\left(f\right)$$

$$PDC_{ij}\left(f\right)=\frac{{\bar{a}}_{ij}\left(f\right)}{\sqrt{\overset{m}{\underset{k=1}{\sum }}{\left|{\bar{a}}_{kj}\left(f\right)\right|}^{2}}}$$

where **I** refers to the identity matrix, ${\bar{a}}_{ij}\left(f\right)$ represents the corresponding element in matrix $\bar{A}\left(f\right)$ , and *PDC_ij_*(*f*) denotes the relative coupling strength of channel *j* to channel *i* compared to its coupling strength to all other channels. The value range of the PDC coefficients is 0–1, the larger the value, the stronger degree of coupling.

Compared to other connectivity measures, PDC is able to detect directed causal relationships and exclude indirect connections (21). In this study, the PDC connectivity coefficients in the entire frequency domain were considered. By calculating the average of the PDC matrices at different frequencies, an asymmetric matrix was obtained, where the coefficients represent the connection strength between different channels. To use the functional connectivity matrix as the matrix of coupling model subsets, the elements on the diagonal of the PDC matrix were set to zero.

### 2.3 Coupled neural mass model

The subset of the coupled NMM adopted the Wendling’s model, which consists of pyramidal neurons and three interneurons. The differential equations of the Wendling’s model are expressed as follows:


$$\left\{\begin{array}{l} y_{0}\dot{(}t)=y_{5}\left(t\right)\\ y_{5}\dot{(}t)=AaS\left[CI+y_{1}\left(t\right)-y_{2}\left(t\right)-y_{3}\left(t\right)\right]-2ay_{5}\left(t\right)-a^{2}y_{0}\left(t\right)\\ y_{1}\dot{(}t)=y_{6}\left(t\right)\\ y_{6}\dot{(}t)=\mathrm{Aa}\left\{p\left(t\right)+C_{2}S\left[C_{1}y_{0}\left(t\right)\right]\right\}-2ay_{6}\left(t\right)-a^{2}y_{1}\left(t\right)\\ y_{2}\dot{(}t)=y_{7}\left(t\right)\\ y_{7}\dot{(}t)=BbC_{4}S\left[C_{3}y_{0}\left(t\right)\right]-2by_{7}\left(t\right)-b^{2}y_{2}\left(t\right)\\ y_{3}\dot{(}t)=y_{8}\left(t\right)\\ y_{8}\dot{(}t)=\mathrm{Gg}C_{7}S\left[C_{5}y_{0}\left(t\right)-C_{6}y_{4}\left(t\right)\right]-2gy_{8}\left(t\right)-g^{2}y_{3}\left(t\right)\\ y_{4}\dot{(}t)=y_{9}\left(t\right)\\ y_{9}\dot{(}t)=BbS\left[C_{3}y_{0}\left(t\right)\right]-2by_{9}\left(t\right)-b^{2}y_{4}\left(t\right) \end{array}\right.$$

Here, $S\left(v\right)=2e_{0}/\left[1+e^{r\left(v_{0}-v\right)}\right]$ . *CI* represents the coupling input, which is determined by the model subpopulation state *y*_1_(*t*) at the past time and the coupling coefficients. $p\left(t\right)$ represents the external perturbation input, introduced as an influence using a Gaussian white noise model. This set of equations can be solved using classical numerical integration methods. The output of the model is the simulated signal $y\left(t\right)=y_{1}\left(t\right)-y_{2}\left(t\right)-y_{3}\left(t\right)$ . The model parameters, their physiological interpretations, and standard values are presented in Supplementary Table S1 (39).

To investigate the brain networks of patients, a Wendling’s model was built for each electrode contact in SEEG and a coupled NMM was constructed. These model subsets were coupled using the PDC matrix described in section 2.2, indicating that the coupling coefficients in the coupled model were based on the elements of the PDC matrix. SEEG differs from EEG in that the number of electrode contacts varies greatly among patients, ranging from a few dozen to more than two hundred. Therefore, to adapt the PDC matrix elements to personalized model construction for different patients, further scaling preprocessing was applied. The scaling coefficients were as follows:


$$\mathrm{scale}\;\mathrm{Factor}=\frac{0.5/\mathrm{numChan}}{\mathrm{meanK}}$$

where *numChan* denotes the number of SEEG signal channels in the patients, and *meanK* represents the mean value of the PDC matrix elements for the patients.

### 2.4 Fitting procedure between coupled NMM and SEEG

The parameters of the model reflect transitions of the patients’ physiological state. This study focused on three excitatory and inhibitory synaptic gains A, B, and G in the model. In the simulation process of the coupled NMM, segments of the patients’ SEEG signals were used as ground truth values to simultaneously fit the simulated signals to the real signals from multiple channels. This is done to identify the parameters of each subset in the coupled NMM. The fitting process employed a genetic algorithm, and the fitness function was as follows:


$$\mathrm{fitness}\;\mathrm{Function}={\left(\mathrm{fit}\;\mathrm{Value}-\mathrm{true}\;\mathrm{value}\right)}^{2}$$

In this study, to investigate the dynamic changes in model parameters, the fitting process was performed in the time domain. The genetic algorithm initialized a population by creating individual parameter sets. Through iterations of the population, including individual selection, mutation, and crossover, the individual parameter set that minimized the fitness function was identified. The iterative process continued until reaching the maximum number of generations. A larger population size allowed convergence in fewer iterations, while a higher number of iterations increased the likelihood of achieving higher convergence accuracy. The parameters for the genetic algorithm in this study are shown in Table 2.

**Table 2:** The interpretations and values (range) of parameters in genetic algorithm.

Parameter	Interpretation	Value (range)
A	Average excitatory synaptic gain	2.5–7
B	Average slow dendritic inhibitory synaptic gain	0–50
G	Average fast somatic inhibitory gain	0–30
popSize	Population size	40
mutationRate	Mutation rate	0.1
crossoverRate	Crossover rate	0.5
maxGeneration	Maximum number of generations	50
mu	Mean of Gaussian distribution for mutation	0
sigma	Standard deviation of Gaussian distribution for mutation	0.1

To evaluate the fitting performance, the identified parameters were used as input parameters for coupled NMM simulations. The fitted simulated signals were then compared with the real SEEG signals. The difference between the fitted signals and the real signals was calculated for all channels of patients. The root mean square error (RMSE) was used to quantify the fitting performance.


$$RMSE_{i}=\sqrt{\frac{1}{n}\overset{n}{\underset{j=1}{\sum }}{\left(\mathrm{SEE}G_{i}\left(j\right)-\mathrm{fitLF}P_{i}\left(j\right)\right)}^{2}}$$

where *RMSE_i_* represents the RMSE between the fitted signal and the real SEEG signal for the channel *i* of the patient, *n* is the length of the signal in the temporal domain, *SEEG_i_*(*j*) represents the signal value of the frame *j* in the SEEG signal for the channel *i*, and *fitLFP_i_*(*j*) represents the signal value of the frame *j* in the fitted signal for the channel *i*.

### 2.5 Simulation after virtual surgery

Since synchronization is a significant triggering factor for epileptic seizures, virtual surgeries were performed using the coupled NMM with personalized parameters established earlier. The aim was to investigate the effects of disrupting certain nodes in the network model on other electrode contacts. To compare the postoperative results of different surgical plans, both clinical and random virtual surgeries were conducted. In the clinical virtual surgery, the model subsets corresponding to EZ contacts in a personalized coupled NMM were removed, based on the EZ channels identified by clinicians during radiofrequency thermocoagulation. In the random virtual surgery, we randomly selected an equal number of channels from the NEZ contacts identified by clinicians and removed the model subsets corresponding to them. The removal of model subsets was to set the corresponding elements in the coupling matrix to zero, including the coupling coefficients of the subset to other subsets, as well as the coupling coefficients of other subsets to the subset. For example, patient 1 was implanted eight depth electrodes into the brain, including electrodes Y, X, S, R, T, U, V, W. Clinicians targeted the left precentral gyrus for radiofrequency thermocoagulation and divided the electrode contacts of Y1–Y6, T1–T7, U1–U9, and V1–V8 into the coagulated region. The clinical virtual surgery removed the model subsets corresponding to the coagulated contacts. The random virtual surgery randomly removed the model subsets corresponding to other electrode contacts, and the number of removed model subsets is the same as the number of coagulated contacts.

The postoperative coupled NMM was simulated, and feature extraction was performed on the simulated signals to represent the state of each channel. Since high-frequency oscillations were suggested as the earliest manifestation of epileptogenic networks during a seizure (16), followed by the occurrence of low-frequency oscillations in the subsequent propagating network, the frequency of simulated signals may reflect the epilepsy-related information of the channel. The spectral feature of the simulated signal channels were extracted, specifically the frequency component with the maximum power in the power spectral density, known as the peak frequency (PF), to represent the signal channel. A higher PF of a channel indicated a higher likelihood of abnormal discharges in that signal channel. Due to greater differences between channels during ictal periods (35), the PF of the signal channels in the segment after seizure onset were extracted.

The mean PF values of the simulated signal channels in the postoperative coupled NMM were calculated for the clinical virtual surgery and the random virtual surgery, respectively. These mean PF values represented the overall state of the patients’ brain network under different surgical plans. The mean PF results of the clinical virtual surgery and the random virtual surgery were compared to assess whether the actual radiofrequency thermocoagulation surgery achieved a good surgical outcome. A lower mean PF of the clinical surgery compared to the random surgery indicated that clinical surgery had a more accurate identification of the EZ channels, resulting in a greater difference in postoperative state compared with the random surgery. Therefore, the difference of mean PF between the two virtual surgeries, ∆MeanPeakFrequency was used as a predictive value to estimate the surgical outcomes of patients. ∆MeanPeakFrequency was defined as follows:


$$\Delta \mathrm{MeanPeakFrequency}=MPF_{\mathrm{clin}}-MPF_{\mathrm{rand}}$$

where *MPF_clin_* represented the mean PF of the clinical virtual surgery, and *MPF_rand_* represented the mean PF of the random virtual surgery. To account for the influence of randomness, the process of virtual surgeries and postoperative NMM simulation for each patient was repeated 20 times.

Since the simulated signals of the coupled NMM with fixed model parameters exhibit spikes with different density (35), the same feature, PF, can be used to reflect the state of the signal channels. Following the same steps, the performance of the coupled NMM without incorporating personalized time-varying parameters in predicting the surgical outcomes of patients was further compared. A set of quantitative metrics were used to compare the predictive performance of the two models. By comparing with the actual prognostic outcomes of patients, the false positive rate and the true positive rate at different thresholds were calculated. By constructing a receiver operating characteristic (ROC) curve, the predictive performance was quantified using the area under the ROC curve (AUC). Additionally, the accuracy, recall, precision, and specificity at the threshold closest to the top-left corner of the ROC curve were calculated as evaluation metrics.

### 2.6 Statistical analysis

Based on the postoperative follow-up results of clinical patient information, 12 patients were divided into the SF group (*n* = 6) and the NSF group (*n* = 6). The PDC coefficients of the patients were classified and statistically analyzed according to the division of channels inside EZ and outside EZ, which was judged by clinicians. The brain region that was not EZ was named as non-EZ (NEZ). The mean PDC coefficients were calculated separately for the coefficients between EZ-EZ channels, coefficients between EZ-NEZ channels, and coefficients between NEZ-NEZ channels. The non-parametric sign test was used to compare the mean PDC coefficients between the three channel groups of the SF group and the NSF group.

To perform statistical analysis on the identified parameters A, B, and G, the data was divided into two segments: pre-onset and post-onset. The mean values of the parameters across all subsets for all patients were compared between the two segments using the sign test. Additionally, the parameters A, B, and G were subjected to the sign test in the SF group and the NSF group. This test compared the mean parameter values between subsets classified as EZ and subsets classified as NEZ according to clinicians. Using the Mann–Whitney *U* test, the ∆MeanPeakFrequency of patients in the SF group and the NSF group were compared. The statistical analyses were performed using the SPSS Statistics 26.0 program (IBM, Armonk, NK, United States), and *p* < 0.05 was considered statistically significant.

## 3 Results

### 3.1 PDC matrix coefficients

**Figure 2:**
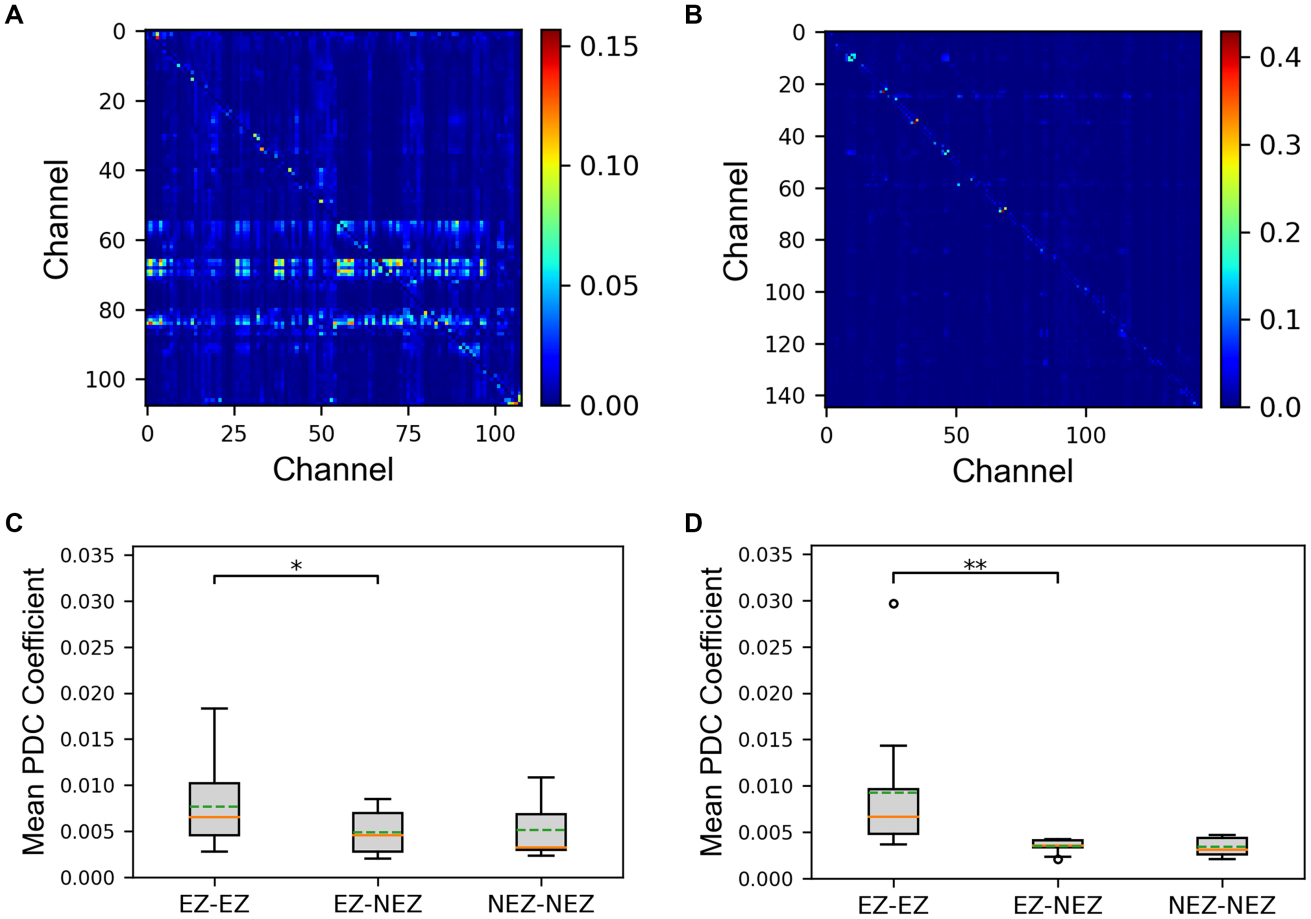
The results of the partial directed coherence (PDC) matrix. **(A)** The matrix plot of an seizure-freedom (SF) patient. **(B)** The matrix plot of an non-seizure-freedom (NSF) patient. **(C)** The comparison of mean PDC coefficients between the SF group. **(D)** The comparison of mean PDC coefficients between the NSF group. The solid lines representing medians and dashed lines representing means. ^*^p = 0.01–0.05, and ^**^p = 0.001–0.01 computed using the non-parametric sign test.

The PDC matrix results of a SF patient and a NSF patient were shown in Figures 2A,B. There was higher connectivity between certain electrode contacts in the patients, and the connectivity was directed, exhibiting significant asymmetry. This may indicate propagation pathways of epilepsy. The mean PDC coefficients results of the SF group and the NSF group were shown in Figures 2C,D. The mean PDC coefficients between EZ-EZ channels in both groups were higher than those between EZ-NEZ channels and NEZ-NEZ channels. The results of the sign test showed that both in the SF and NSF group, the mean PDC coefficients between EZ-EZ channels were significantly higher than that between EZ-NEZ channels (SF group: *p* = 0.039, NSF group: *p* = 0.001). While there was no significant difference observed between other group comparisons.

### 3.2 Fitting and identified parameters of coupled NMM

**Figure 3:**
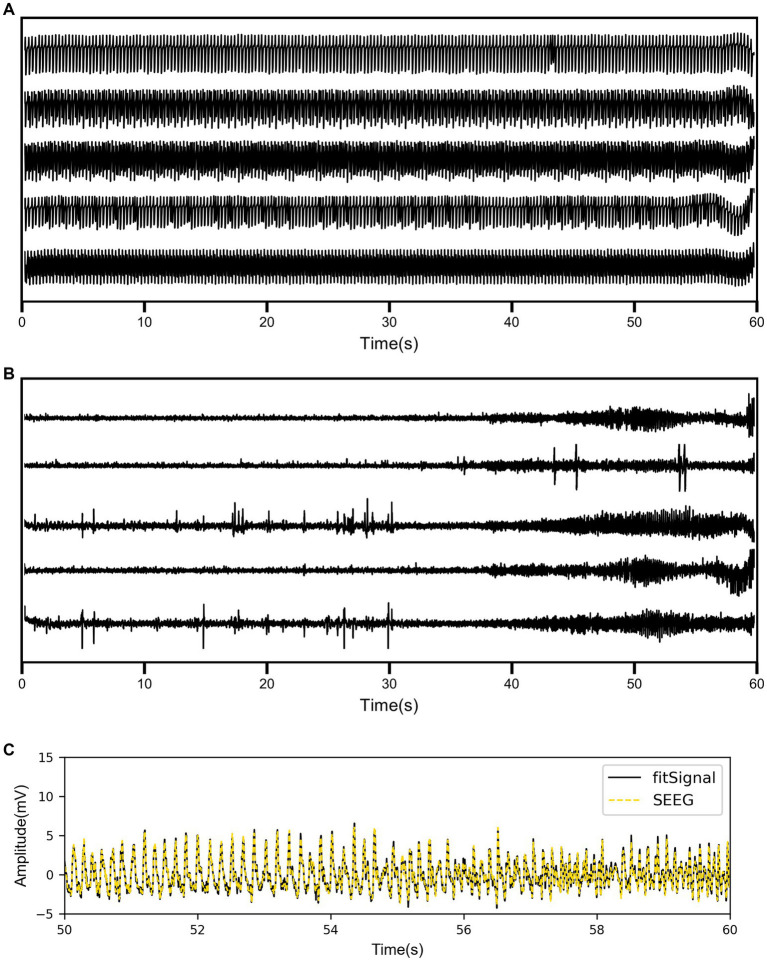
**(A)** The simulation results of coupled neural mass model (NMM) for partial channels before fitting. **(B)** The simulation results of coupled NMM for the same channels after fitting. **(C)** The comparison between the real SEEG signal and the fitted signal for one channel.

Coupled NMM was simulated using the patients’ PDC coefficients, with a fixed set of parameters including three synaptic gains. In Figure 3A, a patient was taken as an example to show the simulation results of partial channels. The simulated signals before fitting exhibited sustained spikes, with potential differences in signal frequencies among different channels. Additionally, simulated signals may exhibit changes in spike density over time, but there were no apparent differences before and after seizure onset.

As shown in Figure 3B, the simulated signal results after fitting displayed the same five channels of the same patient. There were significant differences in the waveforms of different channels, and the signals before and after onset showed distinct variations. Some channels exhibited epileptiform discharges before seizure onset and high-frequency oscillatory discharges after seizure onset. To visually demonstrate the fitting performance of the genetic algorithm, the real SEEG signal and the fitted signal for one of the channels were compared in Figure 3C. The quantified RMSE results for all patients are listed in Table 3. The overall RMSE result was reported as 0.4903 ± 0.2187 mV.

**Table 3:** RMSE between fitted signal and real signal.

Patient	RMSE (mV)	
	Pre-onset	Post-onset
1	0.3532 ± 0.1004	0.3063 ± 0.1380
2	0.3089 ± 0.1226	0.4990 ± 0.4119
3	1.1408 ± 0.4320	0.9170 ± 0.3498
4	0.6387 ± 0.0379	0.6553 ± 0.0752
5	0.2579 ± 0.0575	0.2639 ± 0.0390
6	0.3876 ± 0.0894	0.6914 ± 0.0987
7	0.2418 ± 0.0338	0.2467 ± 0.0328
8	0.3213 ± 0.0306	0.4080 ± 0.1250
9	0.2403 ± 0.0424	0.3642 ± 0.0910
10	0.3471 ± 0.0138	0.3661 ± 0.0214
11	0.3094 ± 0.0489	0.6188 ± 0.2058
12	0.5635 ± 0.0262	0.6078 ± 0.0762

**Figure 4:**
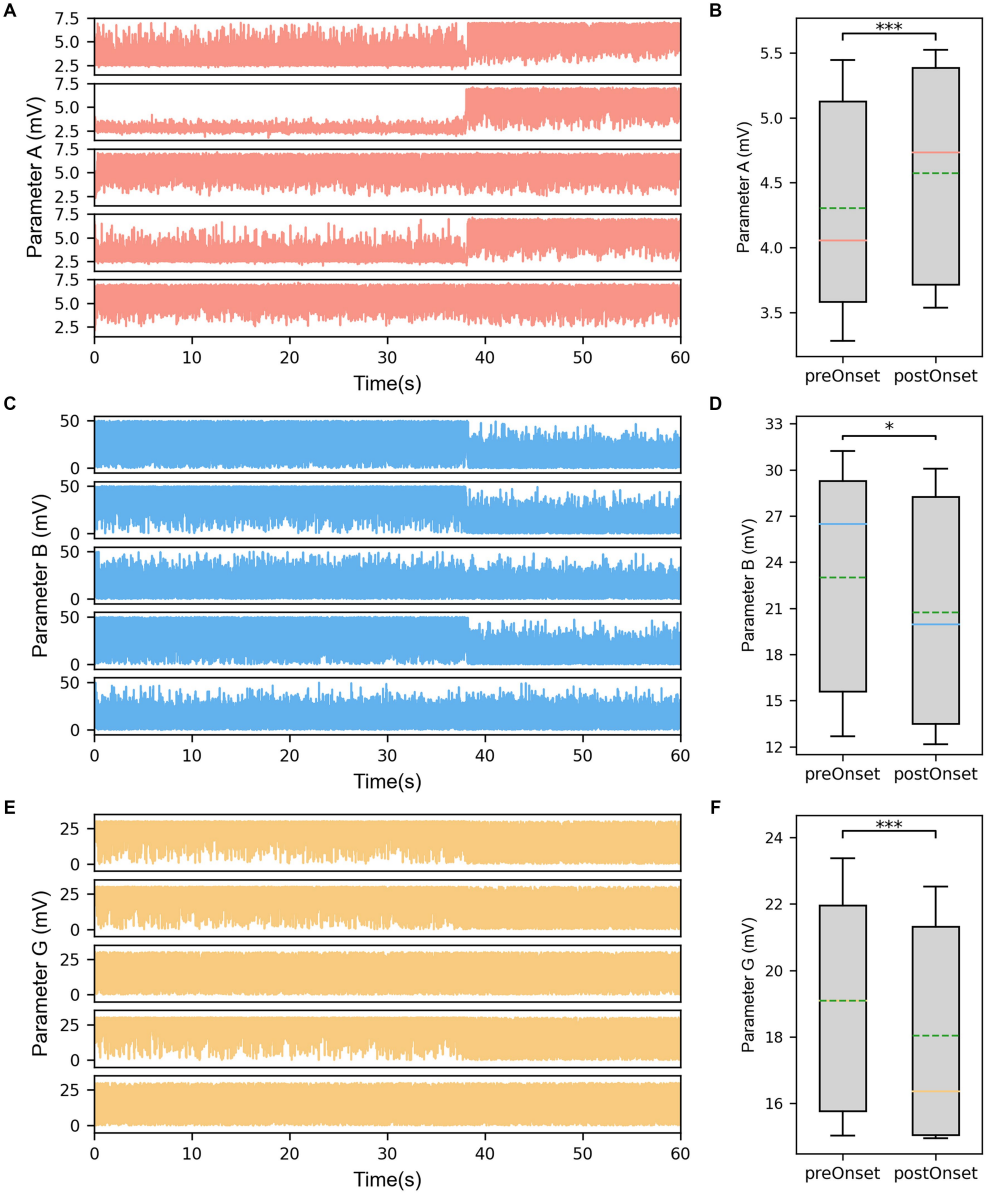
**(A)** The identified parameter sequences A for the same channels. **(B)** The comparison of parameter A before and after seizure onset. **(C)** The identified parameter sequences B for the same channels. **(D)** The comparison of parameter B before and after seizure onset. **(E)** The identified parameter sequences G for the same channels. **(F)** The comparison of parameter G before and after seizure onset. The solid lines representing medians and dashed lines representing means. ^*^p = 0.01–0.05, and ^***^p < 0.001 computed using the non-parametric sign test.

The results displayed the parameter sequences A, B, and G identified by the model subsets corresponding to the same five channels (Figures 4A,C,E). From a temporal perspective, significant differences before and after seizure onset were observed in some subsets of parameters. Statistical test results confirmed that the excitatory parameter A significantly increased after seizure onset (*p* < 0.001, Figure 4B). Conversely, the slow inhibitory parameter B significantly decreased after seizure onset (*p* = 0.035, Figure 4D). Similarly, the fast inhibitory parameter G significantly decreased after seizure onset (*p* < 0.001, Figure 4F).

**Figure 5:**
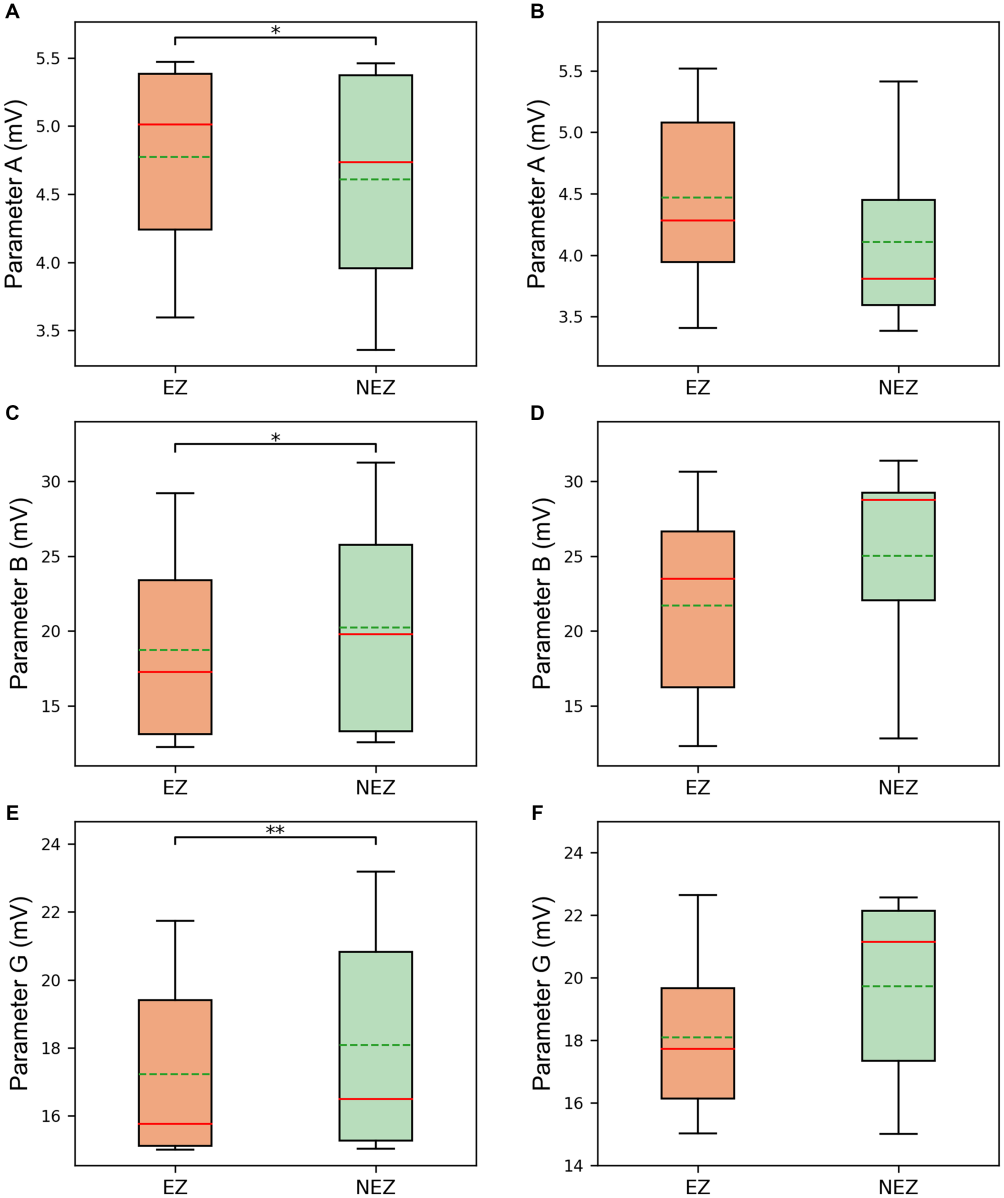
The comparison between EZ subsets and NEZ subsets of **(A)** parameter A in the SF group. **(B)** Parameter A in the NSF group. **(C)** Parameter B in the SF group. **(D)** Parameter B in the NSF group. **(E)** Parameter G in the SF group. **(F)** Parameter G in the NSF group. The solid lines representing medians and dashed lines representing means. ^*^p = 0.01–0.05, and ^**^p = 0.001–0.01 computed using the non-parametric sign test.

Furthermore, it was noted that the parameter values of different subsets at the same time point were also different when comparing the parameter sequences of different subsets. The statistical test results for subset comparisons in the SF group and the NSF group are presented in Figures 5A,C,E and Figures 5B,D,F, respectively. Specifically, the mean value of parameter A for the subsets corresponding to clinically identified EZ channels in the SF group was significantly higher than the mean value of parameter A for the subsets corresponding to clinically identified NEZ channels (*p* = 0.039, Figure 5A). Although the mean value of parameter A for the subsets corresponding to clinically identified EZ channels in the NSF group was also higher than that for the subsets corresponding to clinically identified NEZ channels, the difference was not statistically significant (Figure 5B). Similar test results were obtained for parameters B and G. Parameter B showed a significant difference in the SF group (*p* = 0.039, Figure 5C), while no significant difference was observed in the NSF group (Figure 5D). Parameter G exhibited a significant difference in the SF group (*p* = 0.006, Figure 5E), while no significant difference was observed in the NSF group (Figure 5F).

### 3.3 Predicting surgical outcome using postoperative model

**Figure 6:**
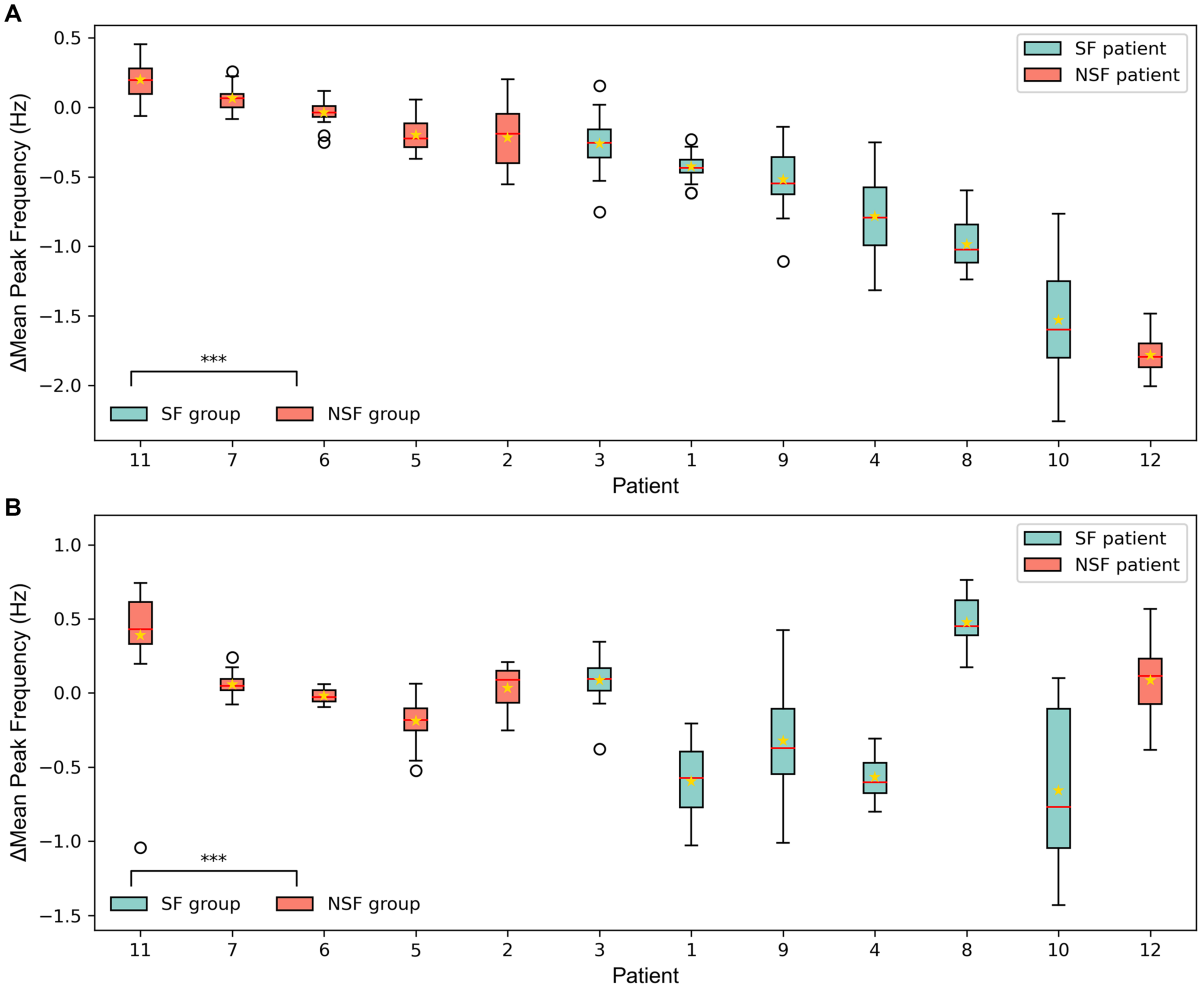
The ∆MeanPeakFrequency of 12 patients from the simulated signal results of the coupled model after the virtual surgery. **(A)** The coupled NMM incorporating personalized dynamic parameters. **(B)** The coupled NMM with fixed parameters set to standard values. The solid lines representing medians and star marks representing means. ^***^p < 0.001 computed using the Mann–Whitney *U* test.

The ∆MeanPeakFrequency calculated from the simulated signals of the coupled NMM after the virtual surgeries for 12 patients was shown in Figure 6. Figures 6A,B displayed the results of the coupled model with personalized time-varying parameters and the fixed-parameter coupled model, respectively. As shown in the bottom left corner of Figures 6A,B, the ∆MeanPeakFrequency of the SF group was significantly lower than that of the NSF group (*p* < 0.001) in both models. Using the ∆MeanPeakFrequency as a classifier for classifying the surgical outcomes of patients, the evaluation metrics for classification performance were shown in Table 4. The coupled model with personalized time-varying parameters achieved an AUC of 83.33% and an accuracy of 91.67%, while the fixed-parameter coupled model showed 69.44 and 83.33%, respectively.

**Table 4:** Quantitative performance of coupled NMM with different model parameters in classifying surgical outcomes of patients.

Evaluation metrics	Classification performance of coupled NMM	
	Personalized parameters	Fixed parameters
AUC	83.33%	69.44%
Threshold	−0.25	−0.25
Accuracy	91.67%	83.33%
Recall	83.33%	100%
Precision	100%	75%
Specificity	100%	100%

## 4 Discussion

This study built personalized coupled models based on NMM, incorporating patient-specific functional connectivity PDC matrices and identified parameters from the patients’ SEEG signals. This study aimed to predict surgical outcomes based on the simulation results using personalized coupled models. It was noted that the computed PDC coefficients exhibited higher values between EZ channels, indicating stronger connectivity between EZ regions. Additionally, a good fit was achieved between the simulated signals and real signals, and parameters consistent with the patients’ physiological characteristics were identified. The dynamics of the three identified parameters aligned with the physiological processes of seizures, and there were differences between the parameters of channels within and outside the EZ. Finally, the personalized coupled model was used for virtual surgery to predict surgical outcomes based on the simulated signals of postoperative models, and the achieved AUC and accuracy demonstrated satisfactory performance.

### 4.1 PDC from SEEG reflects high synchrony within the EZ

It is well known that excessive synchrony among neurons is one of the underlying causes of seizures (36), and the connectivity between nodes in the brain network can reflect the synchrony between nodes to an extent. The PDC coefficients between EZ-EZ channels were higher compared to those between EZ-NEZ channels or between NEZ-NEZ channels. This indicated the presence of high synchrony within EZ. This abnormal high connectivity led to mutual influence and stimulation between these channels, making it easier to reach a seizure state. Similar study findings have been reported by Warren et al. (40), who used average phase coherence as an indicator and found that the seizure onset zone was not connected to other adjacent regions. Bartolomei et al. (16) also suggested that functional connectivity measures indicated the association between epileptic seizures and abnormal synchrony among brain tissue. By coupling NMM subsets with the PDC matrix, the coupled model exhibited a structure that aligns with the epileptic network characteristics of the patient.

### 4.2 Changes in excitation and inhibition dynamic balance during seizures

However, there were still substantial differences between the coupled model and the actual brain network of the patient. Notably, another major factor in epileptic seizures, i.e., the imbalance between excitation and inhibition in the brain, has not been incorporated into this model. Therefore, we believe that the three synaptic gain parameters in the coupled model are crucial and should not be ignored or fixed to standard values (41, 42). To address this problem, a genetic algorithm was employed to minimize the errors between the simulated signals of all channels in the coupled model and the real SEEG signals from the patient, thereby identifying model subset parameters consistent with the patient’s physiological characteristics of each channel. By comparing the simulated signals before and after fitting process, the model’s fitted signals better match the inter-channel differences and temporal dynamic changes observed in the patients’ actual electrophysiological processes. Furthermore, the identified parameters also reflected changes before and after seizure onset. The excitatory parameter A significantly increased after seizure onset, while the slow inhibitory parameter B and fast inhibitory parameter G significantly decreased after seizure onset, demonstrating that the identified parameters can reflect the physiological changes in the brain network during the transition of epileptic seizure activity. Wendling et al. (43) used spectral fitting between a single NMM simulated signal and a real SEEG single-channel signal to identify parameters A, B, and G, and observed an increase in excitatory parameter and a decrease in inhibitory parameters after seizure onset as well. Additionally, in this study, the excitation in the EZ subsets was higher than that in the NEZ subsets, while both inhibitions in the EZ subsets were lower than that in the NEZ subsets. The presence of high excitation and low inhibition reflected the degree to which brain tissue is prone to epileptic seizures, indicating that the identified parameters also correspond to the high epileptogenicity of EZ region.

On the other hand, according to the statistical results, significant differences were found in all three parameters between EZ channels and NEZ channels in the SF group, while in the NSF group, there were no significant differences in parameters A, B, and G. The significant differences in parameters between EZ and NEZ subsets in the SF group may indicate that the clinical judgment of EZ in the SF group was more accurate and can better identify the actual epileptogenic foci with a higher excitatory-inhibitory ratio. However, in the NSF group, the clinical judgment of EZ may be inaccurate, leading to the omission of potential epileptogenic foci, thereby resulting in the absence of significant differences in parameters between EZ and NEZ subsets. Similarly, Sinha et al. (34) found a high consistency between nodes with higher seizure likelihood and resected tissue in patients with good surgical outcomes, whereas there was a lack of consistency in patients with poor surgical outcomes. Moreover, this phenomenon did not exist in the previous statistical analysis of the PDC coefficients, which further demonstrates the value of incorporating personalized parameters into the coupled model.

### 4.3 Simulated signals after virtual surgery to predict surgical outcomes

In this study, the mean PF of the simulated signals from the coupled models was compared under different surgical plans. The majority of patients showed better clinical virtual surgery results compared to random virtual surgery, indicating a more stable overall state of the brain network after clinical virtual surgery. The statistical analysis results showed that the ∆MeanPeakFrequency of the SF group was significantly smaller than that of the NSF group, indicating that the clinical virtual surgery results of SF patients were significantly better than random virtual surgery, while the difference was less pronounced for NSF patients. This may suggest that the EZ localization by clinicians in SF patients was more accurate, resulting in a lower *MPF_clin_* after clinical virtual surgery. Besides, there were fewer potentially missed actual epileptogenic contacts within the NEZ, and the *MPF_rand_* was higher after random virtual surgery conducted in the NEZ. In contrast, the *MPF_rand_* of NSF patients was lower due to less accurate EZ localization and potentially missed actual EZ within the NEZ. Hence, the ∆MeanPeakFrequency between clinical and random virtual surgery was larger for NSF patients compared to SF patients.

The coupled NMM incorporating the identified personalized time-varying parameters, can be used to predict surgical outcomes considering the synchrony, excitation and inhibition of patients’ brain network simultaneously. Compared to the coupled NMM with fixed excitatory and inhibitory parameters, the classification performance of coupled NMM with identified parameters was improved, as indicated by better evaluation metrics. This improvement may be attributed to the fact that in addition to abnormal synchrony, excessive excitability is also a significant trigger for epileptic seizures. Based on the previous discussion, although the subsets of high synchrony were removed, the epileptogenic subsets with high excitation may not be entirely encompassed by the clinicians-defined EZ in NSF patients. Thus the seizure process was not fully disrupted in clinical virtual surgery. On the other hand, for SF patients, the removed EZ subsets identified by clinicians exhibited higher excitation compared to other subsets, resulting in a greater difference between clinical and random virtual surgery. Overall, the proposed method for predicting surgical outcomes has achieved desirable results (34, 44, 45). This analysis is important as it provides a robust method for predicting surgical outcomes, thereby offering significant clinical benefits beyond the initial identification of the EZ. Through the analysis, it is able to predict whether the patients can achieve seizure freedom after surgery. This predictive ability can help clinicians adjust the surgical plans in time, and improve the patient’s recovery rate and quality of life.

### 4.4 Limitations and future work

The sample size of this study was limited, and the robustness of our method should be validated in a larger number of patients in the future. The process of fitting between the SEEG signals and simulated signals from coupled NMM may have the risk of overfitting, and its impact needs to be further considered and avoided. The anti-seizure medication may control the spread of epileptiform EEG activity and affect some functional connectivity measures (46, 47), but its impacts were not considered in this study. Additionally, the temporal dynamic parameters identified by the coupled NMM may be further applied in personalized mechanistic studies of epilepsy seizures for specific patients. In the future, the next step of the research can combine the EZ localization method to validate the effectiveness of different surgical plans, optimizing surgical plans for clinicians.

## 5 Conclusion

This study incorporated personalized parameters into a multi-channel coupled model to establish a brain network model that better aligns with the physiological characteristics of patients. Among 12 focal epilepsy patients, three parameters identified during the fitting of SEEG signals corresponded to the physiological dynamics during epileptic seizures. Additionally, the variations in parameters between the EZ channels and NEZ channels differed in different prognostic groups, which may indicate inaccuracies in clinical EZ localization for the NSF patients. Considering synchrony, excitation and inhibition in patients, virtual surgery was performed on the personalized coupled models and a novel method was developed for preoperatively predicting surgical outcomes. Using the ∆MeanPeakFrequency from the simulated signals of postoperative models as an indicator, surgical outcomes were predicted, showing consistency with actual prognoses.

## Data Availability

The original contributions presented in the study are included in the article/Supplementary material, further inquiries can be directed to the corresponding authors.
