# Supplementary material for: Predicting radiofrequency thermocoagulation surgical outcomes in refractory focal epilepsy patients using functional coupled neural mass model
Source: Front Neurol. 2024 Aug 23;15:1402004. doi: 10.3389/fneur.2024.1402004 (PMC11377261; doi:10.3389/fneur.2024.1402004)
Supplement: Supplementary file 1 [file Table_1.DOCX]

Supplementary Material

**Supplementary** Table 1. The physiological interpretations and standard values of parameters in the Wendling’s model (39).

| Parameter | Physiological interpretation | Standard value |
| --- | --- | --- |
| $A$ | Average excitatory synaptic gain | 4.0 mV |
| $B$ | Average slow dendritic inhibitory synaptic gain | 40 mV |
| $G$ | Average fast somatic inhibitory gain | 20 mV |
| $a$ | Dendritic average time constant in the feedback excitatory loop | 100 s^-1^ |
| $b$ | Dendritic average time constant in the slow feedback inhibitory loop | 50 s^-1^ |
| $g$ | Somatic average time constant in the fast feedback inhibitory loop | 350 s^-1^ |
| $C_{1},C_{2}$ | Average number of synaptic contacts in the excitatory feedback loop | $C_{1}=C$, $C_{2}=0.8C$ |
| $C_{3},C_{4}$ | Average number of synaptic contacts in the slow feedback inhibitory loop | $C_{3}=C_{4}=0.25C$ |
| $C_{5},C_{6}$ | Average number of synaptic contacts in the fast feedback inhibitory loop | $C_{5}=0.1C$, $C_{6}=0.1C$ |
| $C_{7}$ | Average number of synaptic contacts between slow and fast inhibitory interneurons | $C_{7}=0.8C$, $C=135$ |
| $v_{0},e_{0},r$ | Parameters of the sigmoid function | $v_{0}=6$mV, $e_{0}=2.5$ s^-1^, $r=0.56$mV^-1^ |
| $\mu,\sigma$ | Mean and standard deviation of white noise input | $\mu=90$pulses/s,  $\sigma=30$pulses/s |
